# A DirectView focus reduction assay for high-throughput quantification of neutralizing antibodies against influenza A and B viruses

**DOI:** 10.1016/j.crmeth.2026.101479

**Published:** 2026-06-04

**Authors:** Chenchen Feng, Thomas Rowe, Michael Currier, Ryan Dong, Ying Huang, Ginger Atteberry, Li Wang, Masato Hatta, C. Todd Davis, David E. Wentworth, Bin Zhou

**Affiliations:** 1 Influenza Division, National Center for Immunization and Respiratory Diseases, Centers for Disease Control and Prevention, Atlanta, GA 30329, USA; 2 The Paideia School, Atlanta, GA 30307, USA

**Keywords:** influenza, antibody, neutralization, serological surveillance, pandemic preparedness

## Abstract

The focus reduction assay (FRA) is a widely used method in influenza virus research and surveillance to evaluate neutralizing antibodies. However, traditional FRA relies on time-consuming, labor-intensive immunostaining workflows that constrain throughput. Here, we used a fluorogenic neuraminidase (NA) substrate, BTP3-Neu5Ac, to directly visualize viral foci without immunostaining. Foci were captured with a fluorescence plate reader, and neutralizing antibody titers were determined from foci counts across antibody dilutions. This assay, termed DirectView-FRA, was applied to measure the neutralizing activity of antisera and monoclonal antibodies against seasonal A(H1N1)pdm09, A(H3N2), B/Victoria, and B/Yamagata viruses, as well as high pathogenicity avian influenza A(H5N1) viruses. Results were consistent with those of the traditional FRA. By simplifying the workflow, DirectView-FRA enables rapid, high-throughput testing of neutralizing antibodies to support timely public health responses during influenza epidemics and to assess viruses with pandemic potential.

## Highlights

• Simplified focus reduction assay using a fluorogenic neuraminidase substrate
• Direct visualization and quantification of viral foci without immunostaining
• Strong concordance with traditional FRA for influenza A and B viruses
• High-throughput, scalable serological testing for influenza surveillance

## Motivation

Robust and scalable measurement of neutralizing antibodies is critical for influenza surveillance, vaccine development, and outbreak preparedness. Although focus reduction assay (FRA) is widely used to quantify neutralizing antibodies against influenza viruses, its application is limited by labor-intensive immunostaining workflows that restrict throughput. These limitations are particularly challenging when large sample sets must be analyzed during seasonal epidemics or emerging outbreaks caused by high pathogenicity avian influenza viruses. We sought to develop a streamlined assay that preserves the accuracy of FRA while reducing complexity and enabling high-throughput implementation. By leveraging neuraminidase activity as a direct readout of infection, DirectView-FRA addresses these bottlenecks and expands the practical utility of FRA.

## Graphical abstract

**Figure:**
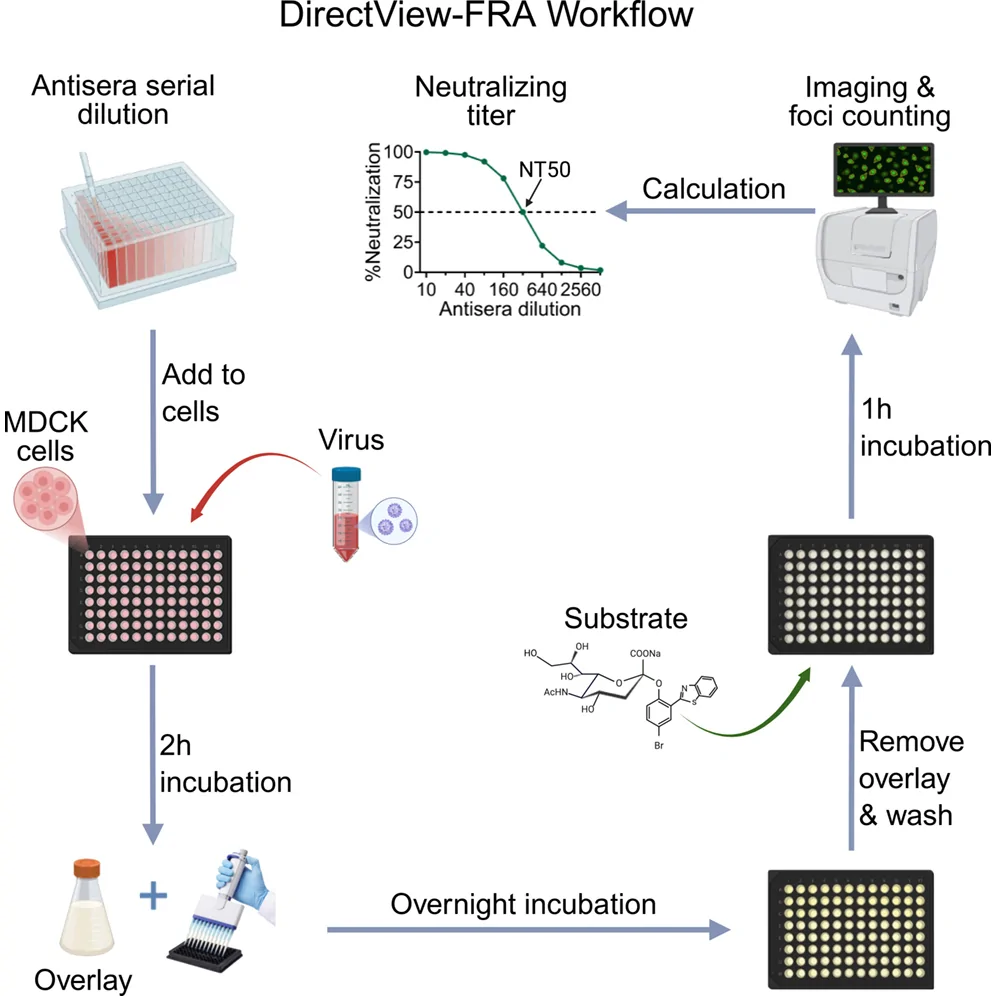

## Teaser

Feng et al. establish DirectView FRA, a high-throughput focus reduction assay that rapidly quantifies influenza-neutralizing antibodies by visualizing viral foci using a fluorogenic neuraminidase substrate. The assay eliminates immunostaining while maintaining accuracy comparable to traditional FRA, enabling large-scale serological analysis for influenza surveillance during epidemics and pandemics.

## Introduction

Seasonal influenza causes up to 650,000 deaths worldwide each year.^1^ In the United States, the disease burden estimates for the 2023–2024 season were 34–75 million influenza cases, resulting in 380,000 to 900,000 hospitalizations.^2^ Additionally, avian influenza, especially high pathogenicity avian influenza A(H5N1), has significant potential for cross-species transmission, including the ability to infect mammals and humans. Between January 2003 and August 2025, there were 990 confirmed cases of human infection with A(H5N1) worldwide and a case fatality proportion of approximately 48%.^3^ Since April 2024, the 2.3.4.4b clade of A(H5N1) virus infected 1,075 dairy cattle farms across 17 states in the United States, and 70 human cases have been confirmed.^4^^,^^5^ Together, seasonal and avian influenza A(H5N1) viruses pose substantial risks to global public health due to seasonal epidemics and pandemic potential.

Vaccination is one of the most effective interventions to mitigate the public health threat of influenza.^6^ A key outcome of vaccination is the elicitation of neutralizing antibodies (nAbs), which inhibit virus replication through multiple mechanisms and targets. Some antibodies target conserved sites within the sialic-acid-binding pocket on the globular head of hemagglutinin (HA), blocking viral binding.^7^^,^^8^ Others bind to the conserved region of the HA stem, preventing membrane fusion.^9^^,^^10^^,^^11^ Additionally, some antibodies target neuraminidase (NA), a second influenza surface glycoprotein, thereby inhibiting the release of progeny virions.^9^^,^^12^ A growing body of research demonstrates that nAb levels correlate with the vaccine-mediated protection against influenza infection.^13^^,^^14^ Therefore, the rapid and reliable evaluation of the virus-neutralizing potential of antisera and antibodies is imperative.

Hemagglutination inhibition (HI) assay, microneutralization (MN) assay, and focus reduction assay (FRA) are the most commonly used tests for measuring inhibiting or neutralizing antibody titers, though each has its own limitations.^15^^,^^16^^,^^17^ The HI assay only measures antibodies that target epitopes on the HA head involved in sialic acid binding to erythrocytes, as only these antibodies can inhibit virus-receptor binding and hemagglutination.^18^^,^^19^^,^^20^ The source of red blood cells also affects the HI results.^21^^,^^22^ The MN assay measures nAbs and typically uses cytopathic effect (CPE) as a readout of virus replication/neutralization, which requires multiple days of infection before CPE can be observed.^21^^,^^23^ In contrast, the FRA uses immunocytochemistry to stain viral foci, offering a more quantitative approach and requiring only a single day of infection to obtain results.^17^^,^^24^ Current FRA methods, however, are not suitable for high-throughput testing of large sample sets due to the time-consuming and labor-intensive steps of fixation, permeabilization, and immunostaining. Therefore, a rapid, high-throughput assay for measuring nAbs is urgently needed.

**Figure 1:**
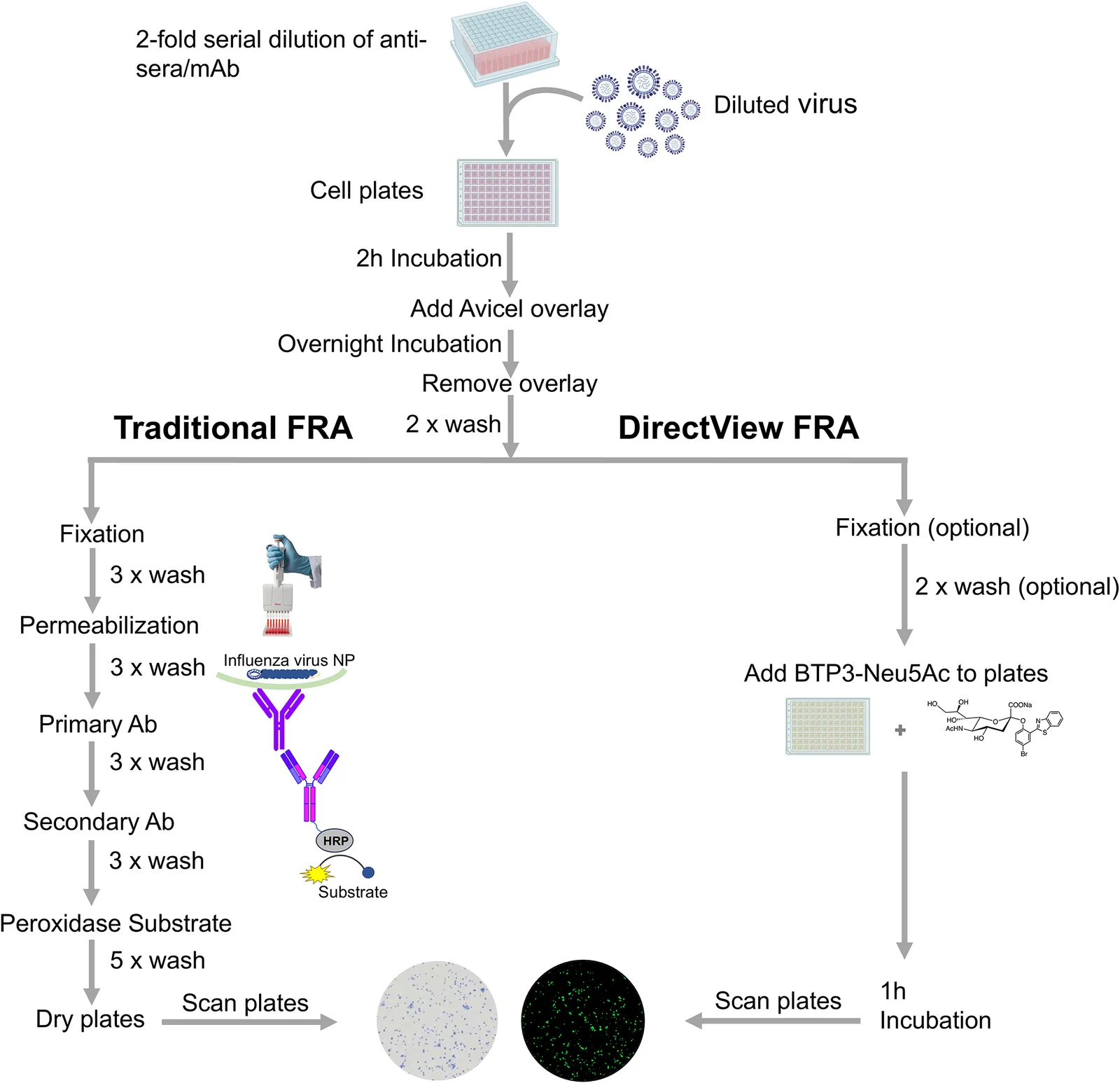
Schematic workflow of traditional FRA and DirectView-FRA for assessment of neutralization antibodies against influenza virus Both assays can be utilized to evaluate neutralizing activity of antibodies against influenza viruses. Instead of using primary antibody and secondary antibody to amplify the signal, DirectView-FRA uses a one-step fluorogenic reporter, BTP3-Neu5Ac, to detect enzymatic activity of neuraminidase molecules on the surface of virus-infected cells. Briefly, serially diluted antiserum was mixed with 600 focus forming units of virus before transferring the mixture to a 96-well MDCK cell plate for 2-h incubation at 37°C. Next, an Avicel overlay was added to the cells and incubated for additional 18–20 h at 37°C for seasonal influenza viruses or 14 h at 32°C for high pathogenicity avian influenza viruses (see method details). After incubation, plates were then washed to remove the overlay, and BTP3-Neu5Ac substrate was added directly to the plates and incubation at 37°C for 1 h. Fluorescent foci were detected using a high-content imaging plate reader. For biosafety consideration, an optional fixation step may be performed prior to substrate addition. In this case, cells were fixed with 70% ethanol for 30 min at 4°C, followed by two washes to remove residual fixative before substrate addition. Compared to the traditional FRA, DirectView-FRA has a simpler process and higher throughput.

In this study, we describe the development and validation of a simplified, rapid FRA for quantifying nAbs (Figure 1). This assay utilizes the fluorogenic neuraminidase substrate, BTP3-Neu5Ac, which, upon cleavage by neuraminidase, generates a fluorescent, water-insoluble product that accumulates on infected cells, forming fluorescent foci.^25^^,^^26^^,^^27^ The fluorescent foci can then be directly quantified using a plate reader, allowing for precise nAb titer determination. The DirectView-FRA produces results comparable to the traditional FRA while offering substantial time savings when testing large numbers of samples, making it suitable for high-throughput applications.

## Results

### Optimization of DirectView-FRA reaction conditions

**Figure 2:**
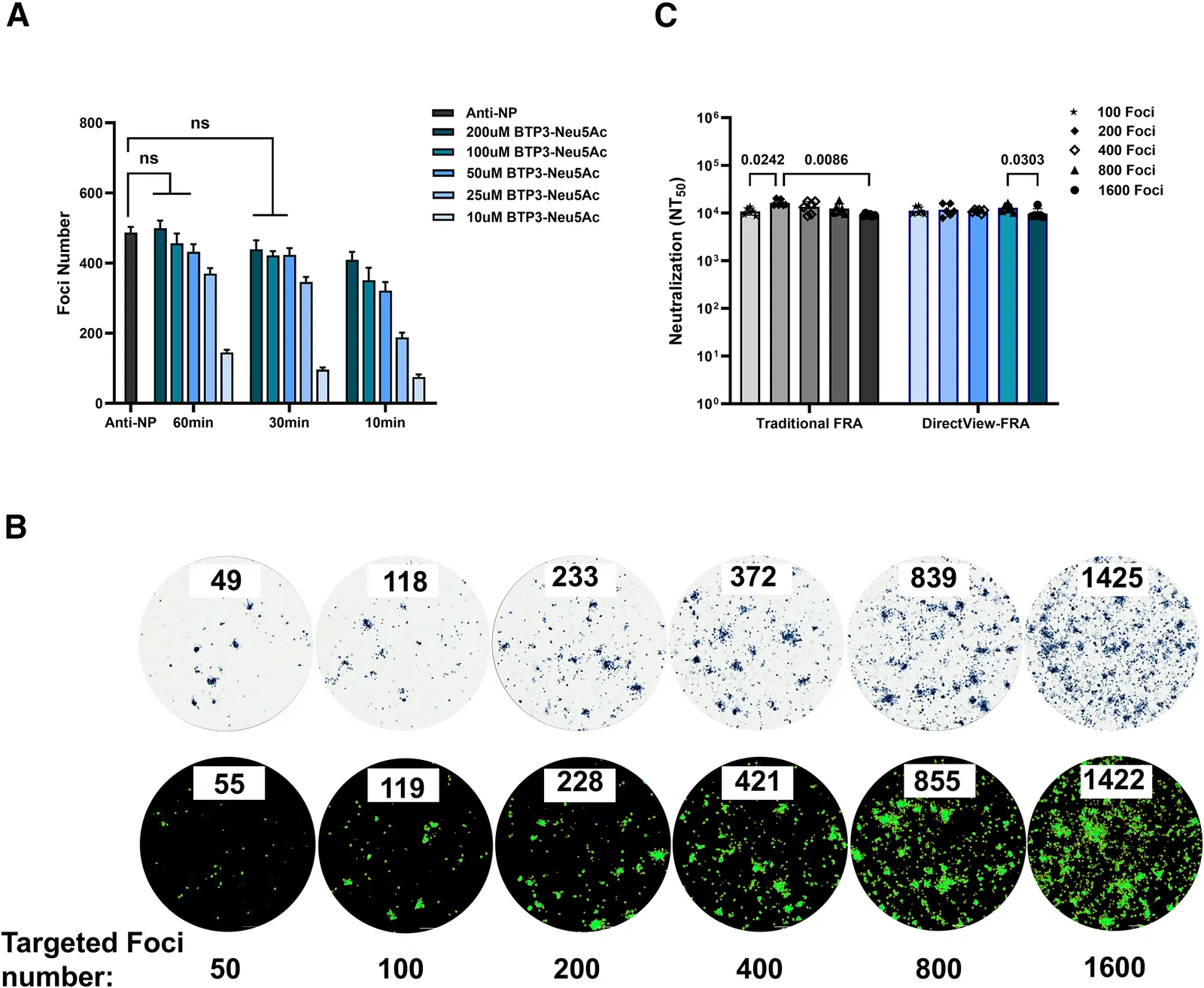
Optimization of reaction conditions in quantification of foci and neutralization titers (A) Sensitivity of BTP3-Neu5Ac at different concentrations with different incubation times in detecting FFU. (B) Representative images of the number of viral foci when the virus was 1:2 serially diluted, and the same volume was added to the traditional FRA and DirectView-FRA wells. Targeted foci numbers are 50, 100, 200, 400, 800, and 1,600. The upper row and bottom row were detected by traditional FRA and DirectView-FRA, respectively, with actual foci number shown on each image. (C) Comparison of neutralization titers of a ferret serum against different input doses of NY/2009 by traditional FRA and DirectView-FRA. Two-way ANOVA with Dunnett’s multiple comparisons test was used for (A), whereas two-way ANOVA with Tukey’s multiple comparisons test was used for (C). In (A), the DirectView-FRA values are compared to traditional FRA, and only the non-significant comparisons are labeled as ns. In (C), all values are compared, and only the significant ones are labeled with *p* value.

To develop and optimize the DirectView-FRA, we tested various working concentrations of the BTP3-Neu5Ac substrate and lengths of reaction time using an A(H1N1)pmd09 strain, A/New York/1682/2009 (NY/2009).^28^ We pre-determined the focus-forming unit (FFU) titer of the virus with a traditional FRA that quantifies the viral foci using anti-NP primary antibody. We then used 500 FFU to infect each well of MDCK cells. After an overnight incubation, the cells were washed, and the foci were stained with anti-NP antibody (traditional FRA) or with BTP3-Neu5Ac (DirectView-FRA) (Figure 1). We tested BTP3-Neu5Ac at concentrations of 200, 100, 50, 25, and 10 μM and assessed incubation times of 60, 30, and 10 min (Figure 2A). Compared to the traditional FRA, the DirectView-FRA produced similar foci counts when 200, 100, and 50 μM of the substrate were incubated for 60 or 30 min (*p* > 0.05). Based on this result, 50-μM substrate concentration and 30-min incubation could be used in the assay; however, we chose 100-μM substrate concentration and 60-min incubation for our subsequent experiments to enhance assay robustness.

To evaluate the working range of the input infectious units (FFU) of DirectView-FRA, we infected cells at various doses of NY/2009, with targeted foci counts ranging from 50 to 1600 (Figure 2B). The results showed that DirectView-FRA could precisely quantify viral foci within a working range of 50–1,600 foci, using traditional FRA as a reference (Figure 2B). We then determined the 50% neutralizing titer (NT_50_) of a homologous ferret antiserum against the NY/2009 virus. DirectView-FRA produced generally consistent NT_50_ titers when the input infectious dose was between 100 and 1,600 foci, which was comparable to the NT_50_ titers from traditional FRA (Figure 2C). Therefore, DirectView-FRA was robust in quantification of nAbs across a wide range of input infectious titers. In subsequent experiments, we targeted 600 foci as the input infectious quantity for neutralization assays.

### Performance of DirectView-FRA with seasonal influenza A and B viruses

**Figure 3:**
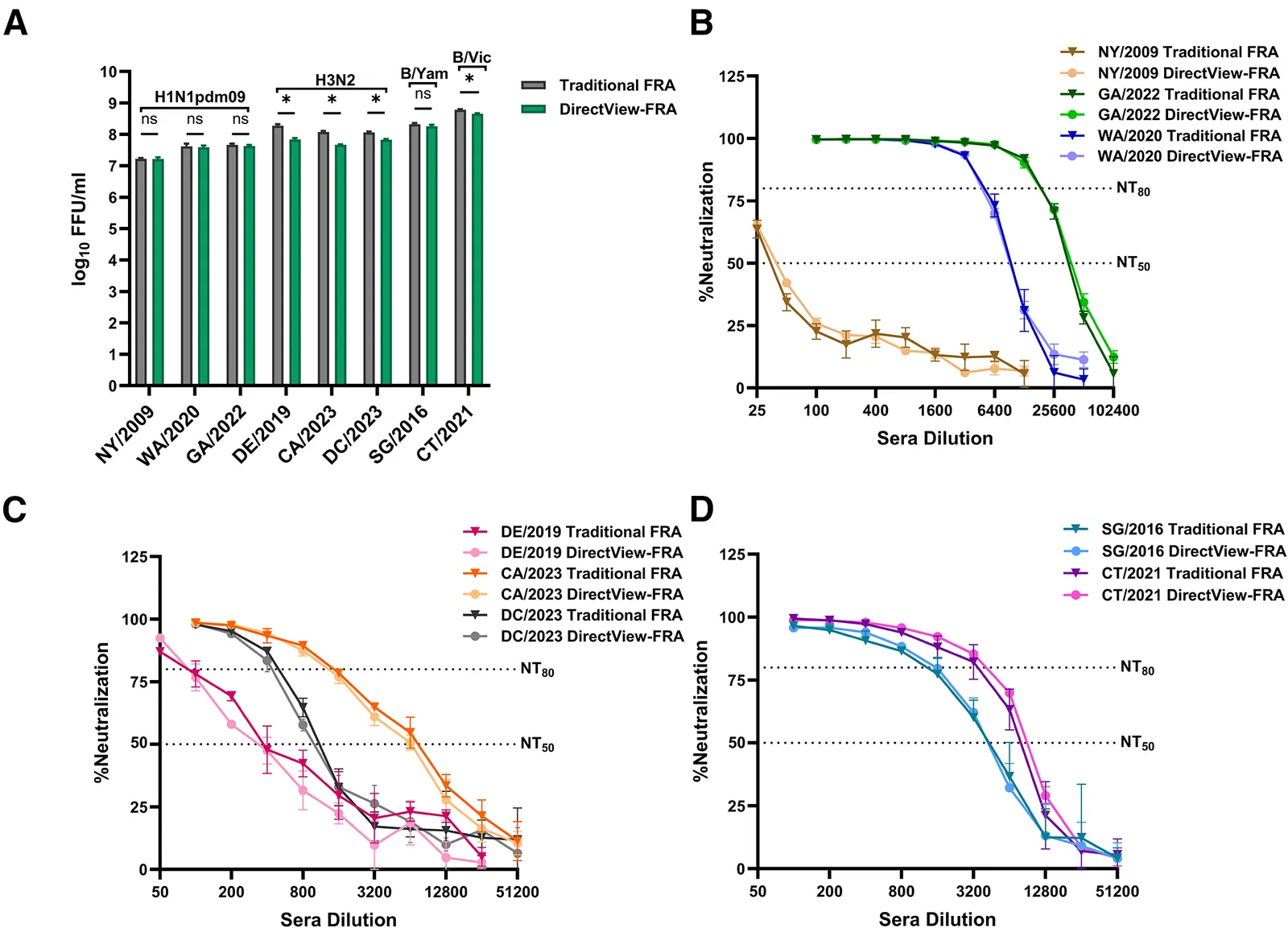
Comparison of traditional FRA and DirectView-FRA for seasonal influenza viruses (A) Titration results of eight seasonal influenza viruses using traditional FRA and DirectView-FRA. The data shown here are viral titers only. (B) Neutralization curves of an A(H1N1)pdm09 ferret antiserum against three A(H1N1)pdm09 viruses. (C) Neutralization curves of an A(H3N2) ferret antiserum against three A(H3N2) viruses. (D) Neutralization curves of a B/Yamagata ferret antiserum against a B/Yamagata virus and a B/Victoria ferret antiserum against a B/Victoria virus. Each data point presents the geometric mean value of three replicates. The values of 50% neutralization (NT_50_) and the 80% neutralization (NT_80_) were marked by the dashed lines and calculated. The data analysis was performed through multiple unpaired *t* test with Welch correction. Asterisks indicate significant difference was observed in DirectView-FRA and traditional FRA; ∗*p* < 0.05, and non-significant “ns” groups also were displayed in (A).

To validate the ability of DirectView-FRA to quantify nAbs against various influenza viruses, we tested the neutralization activity of ferret antisera against a panel of seasonal influenza A and B viruses, using traditional FRA as a comparator. The virus panel comprised eight strains: three A(H1N1)pdm09, three A(H3N2), one B/Victoria, and one B/Yamagata virus. A(H3N2) viruses exhibited 2–3 log lower FFU titers in MDCK cells than in MDCK-SIAT1 cells, whereas the other viruses showed comparable titers across both cell lines (Figure S1). These findings are consistent with previous reports that A(H3N2) viruses depend strongly on human-type α2,6-linked sialic acids, while A(H1N1)pdm09 and influenza B viruses possess broader receptor specificity and replicate efficiently in conventional MDCK cells.^29^^,^^30^ Accordingly, we performed all subsequent assays with seasonal influenza viruses in MDCK-SIAT1 cells. The FFU titers of the viral stocks were determined by both DirectView-FRA and traditional FRA. No significant differences in viral titers were observed between the two assays for the three A(H1N1)pdm09 strains and the B/Yamagata virus (Figure 3A). For A(H3N2) and B/Victoria viruses, although statistically significant, the differences were small (≤0.5 log). For context, titers obtained by plaque assay and TCID_50_ assay differed more substantially from traditional FRA measurements (Table S1).

We used an input dose of 600 FFU in both DirectView FRA and traditional FRA, as determined by their corresponding method, and performed 1:2 serial dilutions of ferret antisera to determine the NT_50_ titers for each virus-serum pair. The overall neutralization patterns and NT_50_ titers detected by DirectView-FRA closely mirrored those detected by traditional FRA (Figures 3B–3D). The NT_50_ titers of the anti-A(H1N1)pdm09 serum (raised against A/Georgia/12/2022 [GA/2022]) against the three A(H1N1)pdm09 viruses, as determined by DirectView-FRA versus traditional FRA, were 45 vs. 38, 9,364 vs. 9,559, and 42,479 vs. 37,520, respectively (Figure 3B). The NT_50_ titers of the anti-A(H3N2) serum (A/California/45/2023 [CA/2023]) against the three A(H3N2) viruses were 364 vs. 529, 5,511 vs. 6,619, and 1,247 vs. 1,277, respectively (Figure 3C). The NT_50_ titers of the homologous anti-B/Yamagata serum (B/Singapore/Inftt-16-0610/2016 [SG/2016]) were 4,019 vs. 4,025 and that of the homologous anti-B/Victoria serum (B/Connecticut/01/2021 [CT/2021]) were 8,723 vs. 7,413 (Figure 3D). Therefore, DirectView-FRA demonstrated equivalence to traditional FRA in quantifying the neutralization activity of antisera and neutralizing antibodies against seasonal influenza.

### Performance of DirectView-FRA with high pathogenicity avian influenza A(H5N1) viruses

Having established the utility of DirectView-FRA in quantifying neutralization activity against seasonal influenza, we extended its application to avian influenza viruses, testing two strains of high pathogenicity avian influenza (HPAI) A(H5N1): A/Chile/25945/2023 (CL/2023) and A/Texas/37/2024 (TX/2024). These strains represented circulating clade 2.3.4.4b in the Americas, with TX/2024 linked to an ongoing epizootic in dairy cattle in the United States. Using both DirectView-FRA and traditional FRA, we evaluated the neutralization activity of six ferret antisera (raised against the HA protein of A/American wigeon/South Carolina/22-000345-001/2021)^31^ and three human monoclonal antibodies (mAbs) against those two human isolates.

**Figure 4:**
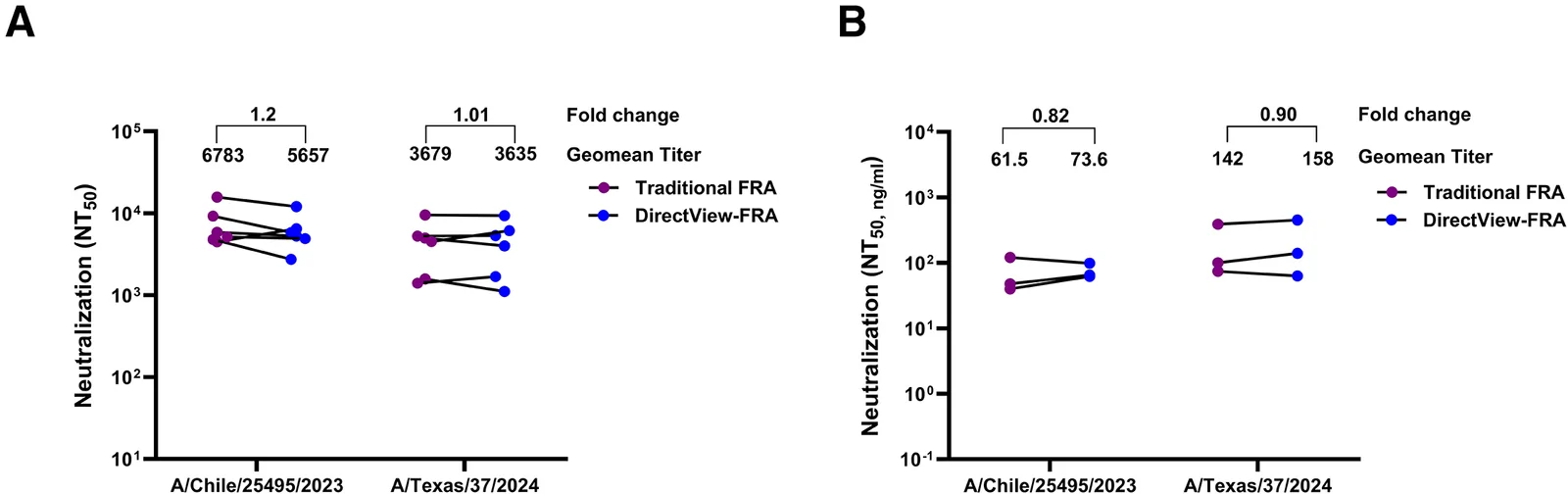
Comparison of traditional FRA and DirectView-FRA for HPAI A(H5N1) viruses (A) Neutralization titers (NT_50_) of six ferret antisera against two A(H5N1) viruses. The *y* axis shows the dilutions made from the original antisera. (B) Neutralization titers of three H5 monoclonal antibodies against two A(H5N1) viruses. The *y* axis shows the concentration of the antibody in ng/mL. The NT_50_ values generated from traditional FRA are shown in purple dots and those from DirectView-FRA are shown in blue dots. The data analysis was performed through multiple paired *t* test comparisons with Wilcoxon test.

For CL/2023, the six ferret antisera showed similar NT_50_ titers across both assays, with each serum exhibiting a non-significant difference of less than 2-fold (*p* > 0.05). The average NT_50_ titer was 5,657 with DirectView-FRA and 6,783 with traditional FRA. Likewise, for TX/2024, individual serum titers varied by less than 2-fold between the two assays (*p* > 0.05), with average titers of 3,635 for DirectView-FRA and 3,679 for traditional FRA (Figure 4A). The NT_50_ values of three mAbs were also consistent between assays, with each mAb exhibiting a non-significant difference of less than 2-fold (*p* > 0.05) (Figure 4B). For CL/2023, the average NT_50_ titer was 73.6 ng/mL with DirectView-FRA and 61.5 ng/mL with traditional FRA; for TX/2024, average NT_50_ titers were 158 ng/mL and 142 ng/mL, respectively.

Together, these results demonstrate that DirectView-FRA reliably quantifies neutralizing activity against HPAI viruses as well as seasonal influenza viruses.

## Discussion

In this study, based on a traditional FRA method, we developed the DirectView-FRA, a streamlined approach that uses a fluorogenic neuraminidase substrate to enable direct visualization of viral foci without immunostaining. This streamlined method not only yielded results consistent with traditional FRA but also offered significant improvements in throughput and labor efficiency, making DirectView-FRA as a versatile and time-efficient tool for evaluating antibody neutralization against various influenza strains, including seasonal and avian viruses.

Using ferret antisera and monoclonal antibodies, we demonstrated that the DirectView-FRA method is highly effective for evaluating neutralizing antibodies against two recent clade 2.3.4.4b A(H5N1) strains. Given that these two HPAI viruses replicate rapidly at 37°C, resulting in overly large foci that hinder reliable quantification after a typical overnight incubation of 18–20 h, we optimized conditions by shortening the incubation period to 14 h and lowering the temperature to 32°C. We anticipate that similar adjustments in incubation time and temperature will also optimize foci size for other HPAI viruses, enabling more accurate quantification.

Since some facilities may only have access to a plate reader for counting fluorescent foci in a BSL-2 lab rather than a BSL-3 lab, we tested fixation of infected cells with 70% ethanol prior to adding the BTP3-Neu5Ac substrate. Viral foci remained clearly visible, and in some cases, more foci were detected after ethanol treatment, likely due to increased substrate accessibility to the neuraminidase proteins. However, to ensure accurate foci quantification, further refinement of the fixation and detection parameters, as well as validation of the protocol, are needed. Additionally, each laboratory must validate complete inactivation of HPAI virus before transferring fixed plates to a BSL-2 lab for foci quantification.

In summary, DirectView-FRA is a simplified, high-throughput neutralization assay that represents a significant advancement over the traditional FRA, offering a scalable, efficient tool to support timely public health responses and accelerate the development of next-generation influenza vaccines and treatments.

### Limitations of the study

DirectView-FRA, similar to the traditional FRA, measures neutralizing antibodies. However, non-neutralizing antibodies can also play a significant role in protection against viral infection through various mechanisms, including antibody-dependent cellular cytotoxicity (ADCC), antibody-dependent cellular phagocytosis (ADCP), or antibody-dependent complement activation.^32^ Appropriate assays are necessary to assess these non-neutralizing antibodies. Another limitation of the DirectView-FRA is that, for optimal sensitivity in detecting the fluorescent product BTP3 in viral foci, a custom filter cube with excitation/emission settings of 372/526 nm is required for use with a plate reader, which may not be available to all plate readers.

## Resource availability

### Lead contact

Further information and requests for resources and reagents should be directed to and will be fulfilled by the lead contact, Bin Zhou (bzhou@cdc.gov).

### Materials availability

This study did not generate new unique reagents.

### Data and code availability

• This study did not generate datasets.
• This paper does not report original code.
• The data generated for this study are available from lead contact upon request.

## Acknowledgments

We thank Jeremy Duke for technical assistance and Trent Bullock for editorial assistance. This study was funded by the US Centers for Disease Control and Prevention (CDC). The findings and conclusions in this report are those of the authors and do not necessarily represent the official position of the US CDC. Use of trade names is for identification only and does not imply endorsement by the US CDC or the US Department of Health and Human Services.

## Author contributions

B.Z. conceived and designed this study. C.F. designed and performed the experiments. T.R., Y.H., L.W., and M.H. performed experiments. C.F., M.C., and R.D. analyzed the data. G.A. contributed the cell-related work. C.F. and B.Z. drafted the manuscript. C.T.D., D.E.W., and B.Z. supervised the study and edited the manuscript. All authors contributed to data interpretation and manuscript revision and approved the final version.

## Declaration of interests

The authors declare no competing interests.

## STAR★Methods

### Key resources table

**Table:** 

REAGENT or RESOURCE	SOURCE	IDENTIFIER
**Antibodies**		

Mouse Monoclonal Antibody Influenza Type A (Pool)	CDC-IRR	FR-1217
Mouse Monoclonal Antibody Influenza Type B (Pool)	CDC-IRR	FR-1218
Anti-Mouse IgG (gamma) Antibody-HRP	SeraCare	Cat# 5220-0460

**Bacterial and virus strains**		

A/New York/1682/2009 (NY/2009)	CDC	N/A
A/Washington/23/2020 (WA/2020)	CDC	N/A
A/Georgia/12/2022 (GA/2022)	CDC	N/A
A/Delaware/39/2019 (DE/2019)	CDC	N/A
A/California/45/2023 (CA/2023)	CDC	N/A
A/District of Columbia/27/2023 (DC/2023)	CDC	N/A
B/Singapore/Inftt-16-0610/2016 (SG/2016)	CDC	N/A
B/Connecticut/2021 (CT/2021)	CDC	N/A
A/Chile/25945/2023 (CL/2023)	CDC	N/A
A/Texas/37/2024 (TX/2024)	CDC	N/A

**Biological samples**		

Ferret antisera against seasonal Influenza viruses	CDC	N/A
Ferret antisera against clade 2.3.4.4 b A(H5N1) viruses	CDC	N/A
H5 monoclonal antibodies	CDC	N/A

**Chemicals, peptides, and recombinant proteins**		

Avicel	FMC Biopolymer	RC-581 NF
BTP3- Neu5Ac Na	TCI	B6529
Trypsin from bovine pancreas, TPCK-treated	Sigma-Aldrich	Cat# T1426
TrueBlue KPL	SeraCare	Cat# 5510-0030
Hydrogen peroxide solution	Sigma-Aldrich	Cat# 216763
Glycine	Sigma-Aldrich	Cat# G7126
Triton X-100	Sigma-Aldrich	Cat# T8787
10% Neutral Buffered Formalin	Azer Scientific	Cat# 22-026-445
Bovine Albumin Fraction V (7.5% solution)	Gibco	Cat# 15260037
Bovine Serum Albumin solution (7.5%)	Sigma-Aldrich	Cat# A8412
Ethanol, 200 Proof (100%)	Fisher Scientific	Cat# A4094
1.3% Crystal violet solution	CDC-DSR	Lot# 162119
Geneticin	Gibco	Cat# 3172506
Penicillin-Streptomycin	Thermo Fisher Scientific	Cat# 15140122

**Experimental models: Cell lines**		

MDCK	ATCC	CCL-34
MDCK-SIAT	International Reagent Resource	FR-1380

**Software and algorithms**		

GraphPad Prism software version 10.4.2	GraphPad Software	https://www.graphpad.com
Gene 5	Agilent	https://www.agilent.com/Gen5
ImmunoCapture software 6.4.87	CTL	https://immunospot.com
Bio RENDER	Bio RENDER	https://www.biorender.com

**Other**		

Bio Tek Cytation 7 Cell Imaging Multimode Reader	Agilent	https://www.agilent.com
BioSpot S5 Macro Analyzer	CTL	https://immunospot.com
BioTek ELx405 Select Deep Well Washer	Agilent	https://www.agilent.com/platewasher
Axiocam 202 mono microscope	ZEISS	N/A
96-well Cell culture/imaging microplate	Agilent	204626–100
96-well Clear Microplate	Corning	Cat# 3598
96-well Clear V-Bottom 2mL deep well plate	Corning	Cat# 3960

### Experimental model and study participant details

#### Biosafety and ethics statement

All experiments involving high pathogenicity avian influenza (HPAI) A(H5N1) viruses were conducted in Biosafety Level 3 enhanced (BSL-3E) laboratory at the U.S. Centers for Disease Control and Prevention (CDC), including enhancements required by the U.S. Department of Agriculture and the Federal Select Agent Program. Personnel performing virus-related work received comprehensive training in biosafety protocols and procedural techniques, with competency assessed prior to laboratory access.

Ferret antisera against seasonal influenza viruses were produced previously as part of the CDC Influenza Division’s surveillance program and antisera against the clade 2.3.4.4 b A(H5N1) were generated by vaccination with HA recombinant protein as described previously.^31^ The H5 monoclonal antibodies were also generated previously in a separate study (Feng et al., manuscript in preparation). No animal work was involved in the present study.

#### Cells and viruses

The Madin-Darby Canine Kidney (MDCK) cell line was obtained from American Type Culture Collection (ATCC) and MDCK-SIAT1 was obtained through International Reagent Resource (IRR). MDCK cells were maintained in Modified Eagle Medium (MEM) supplemented with 5% fetal bovine serum (FBS) (37°C, 90–95% humidity, 5% CO_2_). MDCK-SIAT1 were maintained in Dulbecco’s Modified Eagle Medium (DMEM) supplemented with 10% fetal bovine serum (FBS) and 2% Geneticin (37°C, 90–95% humidity, 5% CO_2_).

A(H1N1)pdm09 strains A/New York/1682/2009 (NY/2009), A/Washington/23/2020 (WA/2020), A/Georgia/12/2022 (GA/2022) were isolated and propagated in MDCK cells. A(H3N2) strains A/Delaware/39/2019 (DE/2019), A/California/45/2023 (CA/2023), A/District of Columbia/27/2023 (DC/2023) were isolated and propagated in MDCK-SIAT1 cells. Influenza B Victoria lineage strain B/Connecticut/2021 (CT/2021) and Yamagata lineage strain B/Singapore/Inftt-16-0610/2016 (SG/2016) were isolated and propagated in MDCK cells. High pathogenicity avian influenza A(H5N1) strains, A/Chile/25945/2023 (CL/2023) and A/Texas/37/2024 (TX/2024), were also isolated and propagated in MDCK cells as both were from human specimens.

### Method details

#### DirectView focus reduction assay (DirectView-FRA)

DirectView-FRA was performed as an efficient, high-throughput modification of the traditional focus reduction assay for detecting influenza virus neutralizing antibodies (Figure 1). Briefly, MDCK cells or derivative cells were plated on 96-well microplates at a density of 2.5 × 10^4^ cells/well. After overnight incubation, the cell monolayer is fully confluent, and the cell cultural medium was replaced by 50 μL per well virus growth medium with TPCK-trypsin “VGMT” (MEM, 0.1% fraction-V bovine serum albumin (BSA), antibiotics (penicillin/streptomycin) and 1 μg/mL TPCK-treated trypsin). Then, 50 μL *1/2*log serial diluted (10^−1^ to 10^−6^) virus was added to the corresponding well of the 96-well MDCK cell plate. After adding virus, the cell plate was incubated for 2 h (37°C, 5% CO_2_) followed by addition of 100 μL overlay (equal volumes of 0.06% Avicel RC/CL in MEM containing 1 μg/mL TPCK-treated trypsin, 0.1% Gibco BSA, and 1% Pen Strep) to each well to facilitate lateral infection by infected cells during 14–20 h of incubation at 37°C or 32°C, depending on the virus subtype. Specifically, seasonal influenza viruses were incubated at 37°C for 18–20 h, whereas high pathogenicity avian influenza viruses were incubated at 32°C for 14 h. Following this incubation, the overlay was removed, and the plate was washed twice with PBS, 50 μL of 100 μM BTP3-Neu5Ac substrate was added to each well. Alternatively, after complete removal of overlay, the infected plate was fixed with 70% ethanol at 4°C for 30 min and washed twice with PBS to remove the fixative residual, then incubated with the BTP3-Neu5Ac Substrate. In both methods, the plate was incubated with substrate for 1 h at 37°C. Fluorescent viral foci (spots) were captured using the Bio Tek Cytation 7 imaging reader (Agilent), equipped with a customized filter cube at Ex/Em = 372/526. Viral titer was quantified as focus forming units per mL (FFU/mL), and 1.2 × 10^4^ FFU/mL viral dilution (600 foci/well) was determined and used in the next neutralization step.

For the neutralization step, monoclonal antibody or RDE-treated antisera were prepared by 2-fold serial dilutions in VGMT using a 96-well, Square V-bottom block. Then, the cell plate was washed with PBS before transferring 50 μL of diluted antiserum/mAb from the corresponding well on the antiserum/mAb preparation plate to the cell plate. After antisera/mAb addition, 50 μl of diluted virus (600 foci/well as described above) was added to each well on the cell plate followed by a 2-h incubation (37°C, 5% CO_2_). Subsequently, 100 μL of overlay was added to each well, and plates were incubated at the same time and temperature used for the viral titration step to ensure accurate foci quantification. The infected cell foci numbers were then determined as described above. Curves of neutralization rate were determined for each serum dilution (in log_2_ values) using GraphPad Prism 10.4.2. We used nonlinear regression to determine the antibody dilution that neutralized 50% (NT_50_) of the virus inoculum without antibody (virus control).^33^^,^^34^^,^^35^ Each antiserum was tested in triplicate. All experiments with HPAI were performed in BSL-3 facilities at CDC.

#### Traditional focus reduction assay (FRA)

FRA has been widely used for detecting influenza virus neutralizing antibodies.^17^ We followed a CDC protocol as previously described.^36^ Virus titration and neutralization steps were identical in both the traditional FRA and DirectView-FRA (Figure 1). However, in the traditional FRA, detection of viral foci requires a multi-step process. Following overnight post-infection, the overlay is removed and cells are washed twice with PBS. Cell plates are sequentially processed through fixation, permeabilization, immunostaining, and visualization for foci quantification. For fixation, post-infection cell plates were treated with 4% cold formalin (100 μL/well, 4°C, 30 min). After fixation, cells were washed 3 times with cold PBS and permeabilized with 5% Triton X-100 in PBS/glycine at room temperature for 20 min before proceeding to 3 times wash with cold PBST. The cells were then immunostained with WHO mouse monoclonal antibody influenza type A or B Pool (International Reagent Resource, Cat No. FR-1217 and Cat No. FR-1218) against influenza A or B nucleoproteins at room temperature for 1 h. After 3 times washing with PBST, the cells were incubated with goat anti-mouse IgG secondary Ab conjugated to horseradish peroxidase (HRP) at room temperature for 1 h. Secondary Ab was then removed, and cells washed (3×, cold PBST) before continuing with the visualization procedure. To visualize infected cell foci, the TrueBlue KPL substrate containing 0.03% H_2_O_2_ was added to the cell plate for 10 min incubation at room temperature before a final wash step (5 ×, H_2_O). Plates were dried and infectious foci were imaged and counted using a CTL Bio Spot Analyzer with ImmunoCapture 6.4.87 software. Throughout our procedure, all wash steps were performed with an automatic plate washer (ELx405, BioTek). Data analysis was performed consistently with the approach described under DirectView-FRA.

#### 50% tissue culture infectious dose assay

The 50% tissue culture infectious dose (TCID_50_) of seasonal influenza viruses were determined using MDCK-SIAT1 cells. Briefly, cells were seeded in 96-well plates and grown overnight to 70–95% confluence. Prior to infection, the cell plates were washed with 180 μL/well of infection medium (DMEM, 0.15% Gibco BSA, 1% Penicillin-Streptomycin) supplemented with 2 μg/mL TPCK-trypsin and 100 μL/well of same medium was added into the plate. Virus samples were serially diluted 10-fold in infection medium supplemented with 2 μg/mL TPCK-trypsin and 100 μL of each dilution was added to multiple replicate wells. Plates were incubated at 34°C with 5% CO_2_ for 3–5 days and monitored for cytopathic effect (CPE). Wells were scored as positive or negative based on the presence of CPE, and TCID_50_ values were calculated using the Reed–Muench method and expressed as TCID_50_/mL.

#### Plaque assay

Titers of the seasonal influenza viruses were also determined by plaque assay using MDCK-SIAT1 cells. Cells were seeded in 6-well plates and grown overnight to 90–100% confluence. Prior to infection, the monolayers were washed twice with 1–2 mL/well of infection medium (DMEM supplemented with 0.3% Sigma BSA, 1% penicillin–streptomycin) supplemented with 2 μg/mL TPCK-trypsin. Virus samples were 10-fold serially diluted in the same medium, and 200 μL of each dilution was added to multiple replicate wells and incubated at 37°C for 1 h for adsorption. The inoculum was then removed, and cells were overlaid with 3 mL of infection medium containing 0.6% Avicel and 2 μg/mL TPCK-trypsin, followed by incubation for 3 days at 37°C with 5% CO_2_. After incubation, 2–3 mL of PBS was added to each well twice to gently remove the overlay, Cells were subsequently fixed with 70% ethanol for 2 min and stained with 1.3% crystal violet (Division of Core Laboratory Services and Response, CDC) for 15 min to visualize plaques. Plaques were counted, and viral titers were calculated as plaque-forming units per milliliter (PFU/mL).

### Quantification and statistical analysis

Results from DirectView-FRA and traditional FRA are reported as NT_50_ and analyzed by two-way ANOVA or paired multiple *t* test with Wilcoxon test as appropriate; P-value cutoff of 0.05 was used to determine statistical significance. All statistical and correlative analyses were performed in GraphPad Prism 10.4.2.

## Supplemental information

Document S1. Figure S1 and Table S1

Document S2. Article plus supplemental information
