# Supplementary material for: A DirectView focus reduction assay for high-throughput quantification of neutralizing antibodies against influenza A and B viruses
Source: Cell Rep Methods. 2026 Jun 4;6(8):101479. doi: 10.1016/j.crmeth.2026.101479 (PMC13494541; doi:10.1016/j.crmeth.2026.101479)
Supplement: Supplementary file 1 [file mmc1.pdf]

Cell Reports Methods, Volume *6*

Supplemental information

A DirectView focus reduction assay for high-throughput quantification of neutralizing antibodies against influenza A and B viruses

Chenchen Feng, Thomas Rowe, Michael Currier, Ryan Dong, Ying Huang, Ginger Atteberry, Li Wang, Masato Hatta, C. Todd Davis, David E. Wentworth, and Bin Zhou

Supplemental information

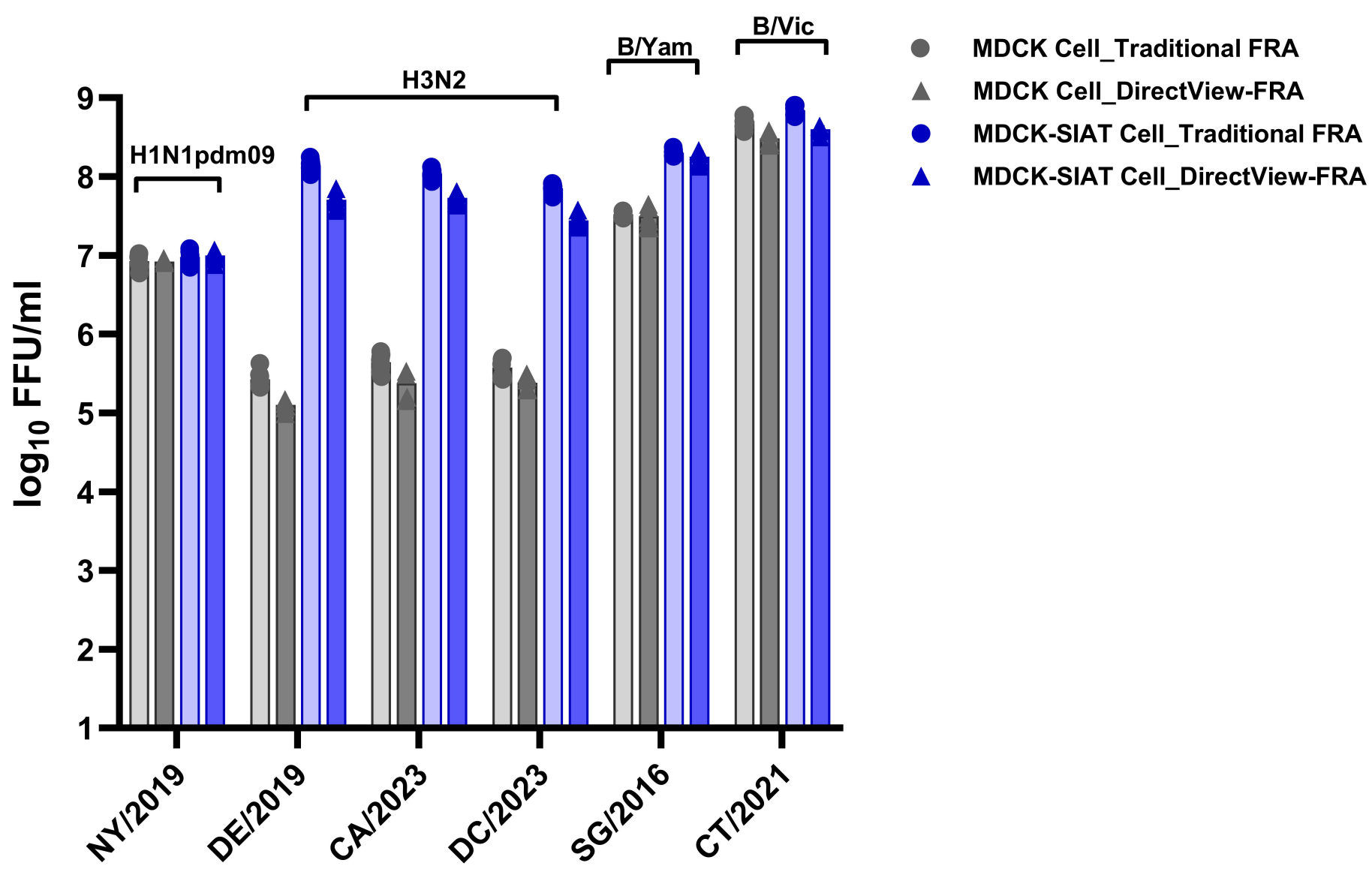

Figure S1. Comparison of viral titers of seasonal influenza viruses in MDCK and MDCK-SIAT1 cells determined by traditional FRA and DirectView-FRA, related to Figure 3. Viral titers of six seasonal influenza viruses were determined in MDCK (gray bars) and MDCK-SIAT1 (blue bars) cells using traditional FRA (circles) and DirectView-FRA (triangles).

Subtype	Strains	Traditional FFU/mL	DirectView FFU/mL	PFU/mL	$TCID_{50}$/mL
H1N1pdm09	A/Georgia/12/2022	7.79 ± 0.10	7.75 ± 0.11	8.18 ± 0.07	8.02 ± 0.37
H3N2	A/District of Columbia/27/2023	7.45 ± 0.17	7.27 ± 0.13	8.16 ± 0.04	7.56 ± 0.24
H3N2	A/Delaware/39/2019	8.25 ± 0.19	7.90 ± 0.22	8.52 ± 0.07	8.08 ± 0.14
H3N2	A/California/45/2023	7.54 ± 0.13	7.12 ± 0.11	8.79 ± 0.05	8.06 ± 0.35
B/Victoria	B/Connecticut/01/2021	8.40 ± 0.07	8.36 ± 0.07	8.86 ± 0.05	7.92 ± 0.43

Table S1. Determination of seasonal influenza virus titers by traditional focus-forming assay, DirectView focus-forming assay, plaque assay, and $TCID_{50}$ assay, related to Figure 3.
